# Vascular Dynamics Aid a Coupled Neurovascular Network Learn Sparse Independent Features: A Computational Model

**DOI:** 10.3389/fncir.2016.00007

**Published:** 2016-02-26

**Authors:** Ryan T. Philips, Karishma Chhabria, V. Srinivasa Chakravarthy

**Affiliations:** Computational Neuroscience Laboratory, Department of Biotechnology, Indian Institute of Technology MadrasChennai, India

**Keywords:** desynchronized vascular dynamics, vasomotion, vascular driven neural computation, neuronal demand, error estimating neurons, predictive coding

## Abstract

Cerebral vascular dynamics are generally thought to be controlled by neural activity in a unidirectional fashion. However, both computational modeling and experimental evidence point to the feedback effects of vascular dynamics on neural activity. Vascular feedback in the form of glucose and oxygen controls neuronal ATP, either directly or via the agency of astrocytes, which in turn modulates neural firing. Recently, a detailed model of the neuron-astrocyte-vessel system has shown how vasomotion can modulate neural firing. Similarly, arguing from known cerebrovascular physiology, an approach known as “hemoneural hypothesis” postulates functional modulation of neural activity by vascular feedback. To instantiate this perspective, we present a computational model in which a network of “vascular units” supplies energy to a neural network. The complex dynamics of the vascular network, modeled by a network of oscillators, turns neurons ON and OFF randomly. The informational consequence of such dynamics is explored in the context of an auto-encoder network. In the proposed model, each vascular unit supplies energy to a subset of hidden neurons of an autoencoder network, which constitutes its “projective field.” Neurons that receive adequate energy in a given trial have reduced threshold, and thus are prone to fire. Dynamics of the vascular network are governed by changes in the reconstruction error of the auto-encoder network, interpreted as the neuronal demand. Vascular feedback causes random inactivation of a subset of hidden neurons in every trial. We observe that, under conditions of desynchronized vascular dynamics, the output reconstruction error is low and the feature vectors learnt are sparse and independent. Our earlier modeling study highlighted the link between desynchronized vascular dynamics and efficient energy delivery in skeletal muscle. We now show that desynchronized vascular dynamics leads to efficient training in an auto-encoder neural network.

## 1. Introduction

Conventionally, information processing in the brain is assumed to be primarily undertaken by neurons, whereas the other constituents (glia, blood vessels) are attributed at best, an auxiliary function. The computational capability of the brain is associated with its large number of individual units (100 billion neurons) and their dense connectivity (order of 10,000 synapses per neuron). Interestingly, recent findings estimate that there are about a 100 billion blood vessels in the human brain (Quaegebeur et al., 2011), suggesting the possibility of vascular regulation at the level of a single neuron.

Blood flow regulation is traditionally thought to be local, in response to the activity of the neurons in the vicinity of the microvasculature (penetrating arterioles and capillaries; Roy and Sherrington, 1890). In general, this is accomplished by the activation of the astrocytes in response to the neurotransmitters (released by the active neurons), which in turn release vasoactive molecules (Zonta et al., 2003; Metea and Newman, 2006). This forms the basis of functional imaging studies such as blood oxygen level dependent (BOLD)-fMRI and positron emission tomography (PET; Horwitz, 2004), although there are studies questioning the criticality of astrocytes in neurovascular coupling (Nizar et al., 2013; Jego et al., 2014). Cortical blood flow could also be directly regulated by innervations from distal neuronal projections as opposed to the conventional view of entirely local regulation (Cohen et al., 1997; Iadecola, 1998; Krimer et al., 1998).

However, neither of these regulations were attributed a role in information processing in the brain. A recent review by Moore and Cao (2008), proposed an active role of the vascular system in governing the excitability of the neurons in the brain. In experiments performed by Sirotin and Das (2009), the hemodynamic signal was observed prior to the neuronal activity before the onset of the next trial, suggesting an anticipatory feedforward role of the vascular system. They further clarify, in response to critique by Kleinschmidt and Müller (2010) and Handwerker and Bandettini (2011), that this hemodynamic signal could be due to neuro-modulatory control of blood vessels (Das and Sirotin, 2011). The decorrelation between hyperemia and neuronal oxygen demand (Leithner and Royl, 2014) further empirically supports the hypothesis (Moore and Cao, 2008) that the vascular system may play an important role in computation in the brain.

Recent theoretical studies (Chander and Chakravarthy, 2012) that simulate a biophysical model of a neuro-glio-vascular unit, also support this hypothesis. This model proposes bidirectional interactions within the neuro-glio-vascular unit: neural activity is communicated to the vascular unit via the glial interface, which in turn receives the energy substrates from the vascular system and passes them on to the neuron, thereby completing the loop. Interestingly, the model also proposes that vasomotion can independently influence neural firing patterns.

If the vascular system can modulate neural activity, the vascular dynamics and vessel to neuron projections take on additional importance. Capillaries constitute approximately 90% of the blood vessels in the brain (Prioreschi, 1996; Viale, 2006), and form a mesh-like structure. Capillaries interact with each other through chemical signals called vaso-mediators (Intaglietta, 1990; Vanhoutte and Mombouli, 1996). These vaso-mediators are released by the endothelial cells, lining the capillaries and govern the overall dynamics of the network. These dynamics can be either chaotic or regular, depending on the vaso-mediators. The transition from regular to chaotic could be a means to enhance tissue perfusion levels (Parthimos et al., 1996). Capillary dynamics affect the neural firing by providing requisite energy “resources” (glucose, lactate, oxygen).

In the present study, information processing in a vascular coupled auto-encoder network is postulated. An auto-encoder could be considered as a model implementing predictive coding (Rao and Ballard, 1999), assuming the response of neurons in the output layer to be predictive of the input given to the network. The prediction of the output layer is accurate when the input reconstruction error is minimized. Thus, there are two sets of neurons, as in the predictive coding framework: the output neurons which act as the predictive units, and the error estimating neurons which estimate the error between the predicted and the actual outputs. The collective activity of these error estimating neurons are conventionally used to train the weights and the biases associated with the network using the back-propagation or the re-circulation algorithm (Hinton and McClelland, 1988). In the present model, the influence of the activity of these error estimating neurons on the activity of the vascular network is modeled.

Autoencoders are based on the paradigm of unsupervised learning wherein the input patterns themselves form the desired/target patterns. A simple linear autoencoder can learn to perform feature extraction or input compression with the network architecture having fewer hidden layer neurons as compared to the input layer neurons. With such a bottleneck, an autoencoder performs dimensionality reduction, extracting the principal components. An autoencoder could also extract interesting features by imposing a sparsity constraint on the hidden layer neurons. Interestingly, these sparsity constraints also yield independent components as described in the seminal work by Olshausen and Field (1996). Now, Independent Component Analysis (ICA) is important for source reconstruction and a number of unsupervised models could also yield the same effect. The underlying feature of such models is an attempt to decorrelate the activity of neurons in the intermediate layers. This could be achieved by introducing lateral connections among the neurons in the intermediate layer and training the connection weights using an anti-hebbian learning rule (Földiak, 1990). Similarly, an algorithm known to implement non-linear-PCA, using a weight normalization term similar to the Sangers rule with a non-linearity introduced, is known to yield independent components (Oja, 1997). However, it is necessary to whiten the input data for the algorithm to work.

Another such regularization constraint, the dropout of neurons in the hidden layer, proposed by Srivastava et al. (2014) has been implemented in the autoencoder model described in the present study. Dropout refers to the random switching off of a certain fraction of neurons in the hidden layer of a neural network during each training iteration. The motivation behind the dropout algorithm is to minimize overfitting on the training data in a supervised learning paradigm. Dropout aids this process by preventing the co-adaption of features learnt by the neurons in the intermediate layers. In other words, the features learnt by individual neurons in the hidden layer are roughly distinct to the features learnt by any other neuron in the hidden layer. This was verified by Srivastava et al. (2014) on considering an unsupervised implementation of the dropout algorithm in an autoencoder. In the auto-encoder context, as opposed to the supervised learning context, the mean squared error(*mse*) (reconstruction error) gives a good estimate of the performance of the network. Using the *mse* in a supervised context would be inappropriate; since the training *mse* may not be reflective of the test performance (measured as a percentage of test patterns correctly classified, on comparing with their corresponding class labels). In an unsupervised auto-encoder, the goal of the network is to learn non-redundant features which will be useful in reconstructing the input pattern. Thus, by definition, the mean squared error, a reconstruction error measure, would be minimized when features ideal for reconstruction, are learnt. These ideal features (which minimize *mse*), are empirically shown to be either purely (in the case bar patterns) or roughly (in the case of MNIST) independent of each other, when the network is trained using the appropriate percentage of dropout. This now raises an important issue, which the authors believe is the main contribution of this paper: Is there a plausible mechanism by which an auxiliary vascular network can regulate the percentage of random dropout in the hidden layer?

Previous studies with autoencoder neural networks have shown that implementing a fixed dropout of hidden neurons improves generalization (Baldi and Sadowski, 2014). In this paper, a mechanism involving a neurovascular loop that implements an optimized version of such a dropout is proposed. We concede that it is possible to envision purely neural sources that could drive such dropout. There are indeed various sources of neural noise such as thermal noise, stochastic molecular diffusion, crosstalk noise, synaptic neurotransmitter release, short term plasticity, ion channel gating etc. (McDonnell and Ward, 2011). But it is not clear if such noise can be modulated by error and used in an optimal fashion to control dropout.

We hypothesize an error feedback loop, partly neural and partly vascular, for driving the dropout. The hemodynamics regulates the firing ability of neurons in the hidden layer and thus incorporates a form of homeostasis. The proposed mechanism has as its basis the following elements from experimental and computational neurobiology:

- In terms of its role in information processing in the brain, neuromodulators such as dopamine have been linked to prediction error (Iadecola, 1998; Schultz, 1998; Schultz and Dickinson, 2000; Steinberg et al., 2013).
- But these neuromodulators also have a vascular aspect to their function: dopaminergic projections to distal vessels are known to control the dynamics of such vessels (Iadecola, 1998; Krimer et al., 1998).
- Vascular rhythms: Vessels exhibits complex rhythms both chaotic and periodic (Nilsson and Aalkjaer, 2003). Temporal chaos in vascular dynamics has been associated with efficient energy delivery (Griffith, 1996). Desynchronized vasomotor oscillations have also been linked to efficient energy delivery in a computational model of perfusion in skeletal muscle (Pradhan et al., 2007). In a similar fashion, given that vascular energy resources in the brain are limited (total blood flow is constrained), the vascular system needs to optimize its supply and not merely cater to the need of all the neurons active (demanding energy) at a particular time instant. Thus, resorting to a desynchronized pattern of supply to the neural tissue is a more feasible strategy employed by the vascular system.
- Zenke et al. (2013) studied possible mechanisms of regulation of firing rates in a cortical model with plasticity, and showed that a feedback homeostatic mechanism, that keeps the firing rates from exploding or from collapsing to zero, must be acting at a time scale of minutes. It is interesting that the vasomotor rhythms are slow operating in the range of a few cycles per minute (Intaglietta, 1990), thereby suggesting a plausible role for vascular dynamics in regulating neural activity.
- A recent adjoining study from our group proposed a low-dimensional neuron-energy model that describes the effect of energy/ATP (outcome of vascular feedback) on neural dynamics. In this work, a rate-coded neuron model in which the threshold of firing depends on ATP, is shown to exhibit dynamics that is nearly identical to a conductance-based neuron model in which ATP is consumed to drive the activity of Na-K-ATPase and K-ATP channel (Chhabria and Chakravarthy, 2016). This study corroborates the notion of ATP modulating the neural firing and also proposes that the variations in the intraneuronal ATP is a resultant of vascular dynamics, thus providing a crucial link between vascular dynamics and neural firing.

**Figure 1:**
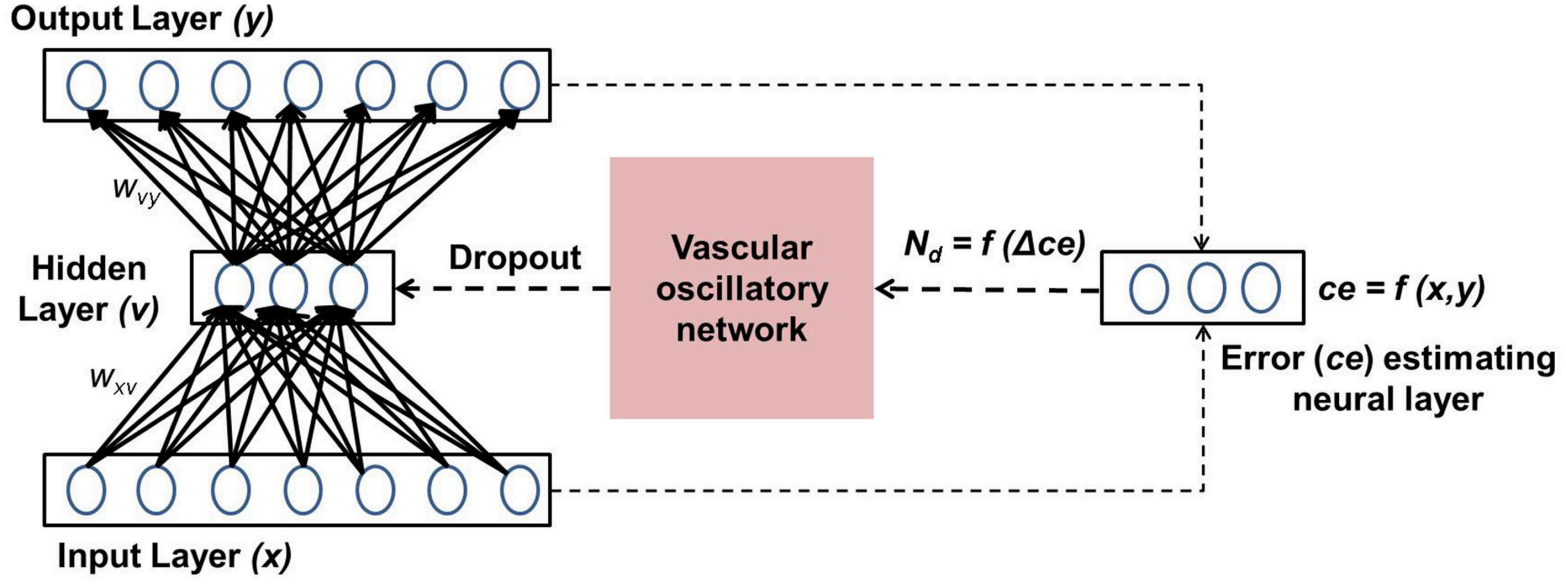
**Schematic representation of the coupled neurovascular model**.

The present model is designed by integrating the above five phenomena in a single framework. The prediction error of the autoencoder (“Error estimating neural layer” in Figure 1) is thought to represent the neuromodulatory system. The error signal thus generated controls vascular dynamics in “Vascular oscillatory network.” Assuming that the feed forward signal to the vascular system comes from the error estimating neurons, as described earlier, it seems logical that the vascular network would now attempt to optimize the supply to the neural system, resulting in optimizing the dropout in the neural network. The output of the vascular oscillators specifically controls the threshold of neural firing in the hidden layer of the autoencoder, thereby controlling dropout.

## 2. Methods

In this section, the important components of the neuro-vascular model are described. The overall model consists of two modules: one representative of the neuronal network, the other of the vascular network. The neuronal network feeds the reconstruction error to the vascular network, whose dynamics enforce a certain percentage of neurons to be OFF in the hidden layer via a feed-back mechanism. A schematic representation of the model architecture is shown in Figure 1.

### 2.1. Neuronal network model

The neural system is modeled as an auto-encoder. An auto-encoder is a multilayer perceptron (MLP), designed such that the activity at the input layer is replicated at the output layer. As in the standard MLP framework there is a hidden layer of neurons, and the entire network is trained using the back propagation algorithm (Oh, 1997), updating weights by stochastic gradient descent on mini batches. Thus, given a neural network with the input vector defined as **x**, the reconstructed input is given as **y**, the activity of a neuron (*v*_*i*_) in the hidden layer is given by Equation 2.

(1) $$\begin{array}{l} h_{i}=w_{i}^{T}x+b_{i} \end{array}$$

(2) $$\begin{array}{l} v_{i}=f\left(h_{i}\right) \end{array}$$

(3) $$\begin{array}{l} f\left(h_{i}\right)=\left\{ \begin{array}{cc} h_{i}\; & :h_{i}>0\\ 0\; & :h_{i}<0 \end{array}\right. \end{array}$$

where **w**_*i*_, *b*_*i*_ correspond to the weight vector that connects the input **x** to the *i*th hidden unit and its bias, respectively. The hidden neurons are linear rectified units, given in Equation 3. The activity of a neuron *y*_*m*_ in the output layer is given as:

(4) $$\begin{array}{l} y_{m}=f\left(\zeta_{m}^{T}v+B_{m}\right) \end{array}$$

where **ζ**_*m*_, *B*_*m*_ correspond to the weight vector that connects the hidden neurons **v** to the *m*th output unit and its bias, respectively. The number of neurons in the hidden layer are chosen such that there is a bottle-neck created between the input and output layers, so as to learn a dimensionally reduced representation of the given input. Traditionally, if a sparse representation of the input is desired, an additional sparsity constraint is imposed. Once sparsity is assured, the weight vectors corresponding to each of the hidden neurons resemble independent components of the input data. Another way in which sparsity of representation can be achieved is by randomly switching off a certain percentage of neurons (*q*) in the hidden layer during training. For each training instance, a different set of hidden neurons are randomly turned OFF. Thus, the activity vector of the hidden layer (*v*^*new*^) is now given by Equation 6.

(5) $$\begin{array}{l} r_{i}\sim \text{Bernoulli(}p\text{)} \end{array}$$

(6) $$\begin{array}{l} v^{new}=v*r \end{array}$$

where the Bernoulli distribution is a discrete distribution having two possible outcomes: *o* = 1 (“success”) occurring with probability *p* and *o* = 0 (“failure”) occurring with probability *q* = 1 − *p*, where 0 < *p* < 1. Thus, *r*_*i*_ determines the state (ON/OFF) of each neuron in the hidden layer. Now, the new activity of each output neuron ($y_{m}^{new}$) is given as:

(7) $$\begin{array}{l} y_{m}^{new}=f\left(\zeta_{m}^{T}v^{new}+B_{m}\right) \end{array}$$

This technique, known as dropout, prevents co-adaptation of the hidden units. Again, the features learnt resemble independent components of the input. The percentage of dropout required is arbitrarily fixed: if too small a percentage is chosen, the co-adaptations would increase, if too large a percentage is chosen, the individual components would not be comprehensive enough to representatively encode the input data in its entirety.

In this paper, it is hypothesized that the neuronal reconstruction error, in this case the mean squared error (*mse*), could influence the percentage of dropout via the vascular network. The mean squared error (*mse*) is defined as:

(8) $$\begin{array}{l} mse=\frac{1}{2*bt*M}\underset{P}{\sum }\underset{m}{\sum }{\left(x_{m}-y_{m}^{new}\right)}^{2} \end{array}$$

where *P* denotes the index of the pattern in a batch of size *bt* presented to the network and *m* denotes the index of a node in the input and output layers respectively of a total of *M* nodes. The desynchronized vascular dynamics itself, allows for random switching OFF of that percentage of neurons in the hidden layer, thereby realizing sparse independent features. Thus, in the proposed model *r*_*i*_ is governed by the vascular network and is further elaborated in Section 2.3.

### 2.2. Vascular network model

The vascular network model is adapted from Pradhan and Chakravarthy (2007) and consists of *n* units. Each of these units are described by non-linear dynamics as given by Equations 9–11.

(9) $$\begin{array}{l} \frac{dg_{j}}{dt}=-g_{j}-u_{j}+\overset{n}{\underset{k=1}{\sum }}T_{jk}S_{k}+I \end{array}$$

and

(10) $$\begin{array}{l} \tau_{v}\frac{du_{j}}{dt}=-u_{j}+S_{j} \end{array}$$

where

(11) $$\begin{array}{l} S_{j}=tanh\left(\lambda_{v}g_{j}\right) \end{array}$$

where *S*_*j*_ denotes the state of the *j*th vessel (ON = +1, OFF = −1) representative of the levels of perfusion; where *S*_*j*_ = +1, and *S*_*j*_ = −1 denote the maximal and minimal perfusion, respectively. It is *S*_*j*_ that influences the neural activity. Furthermore, *g* is a supporting variable and *u* is an auxiliary variable that represents the history of the vessel activity. The slope of the non-linear function in Equation 11 is given by λ_*v*_ and τ_*v*_ is the time constant of the vessel dynamics. The vessels are mutually coupled, in a ring structure with weights described by the *T*_*jk*_ matrix, given by Equation 12. If we assume that there is no coupling between vessel (i.e., *T*_*jk*_ = 0), then if *g* is at a negative (positive) state, a large negative (positive) *u* flips *g* to its positive (negative) state as per Equation 9. Similarly as per Equation 10, *u* simply follows *S* with a delay. A proof that a limit cycle exists in such a configuration is given in Devarajan et al. (2006).

(12) $$\begin{array}{l} T_{jk}=\left\{ \begin{array}{ll} \epsilon -2\exp\left(-d_{jk}∕\sigma^{2}\right)\; & :d_{jk}<3\sigma \\ 0\; & :d_{jk}>3\sigma \end{array}\right. \end{array}$$

(13) $$\begin{array}{l} d_{jk}=\rho {\left[{\left(\cos\left(\alpha_{j}\right)-\cos\left(\alpha_{k}\right)\right)}^{2}+{\left(\sin\left(\alpha_{j}\right)-\sin\left(\alpha_{k}\right)\right)}^{2}\right]}^{1∕2} \end{array}$$

where, ϵ is a crucial model parameter that determines the characteristic dynamics of the vascular network and can have values between 0 and 2, the extremes representing desynchronized and synchronized activities, respectively. While ϵ > 0 denotes that the nearby vessels are inhibitory and distant ones are excitatory, ϵ = 0 represents mutual inhibitory interactions amongst all the vessels. Hence, ϵ controls the balance between excitation and inhibition in the vascular network. The position of the *j*th and the *k*th vessel on the vascular ring are represented by angles α_*j*_ and α_*k*_, respectively. ρ is the distance of any vessel from the neural tissue. The output from the neural tissue modulates the vascular tissue activity, such that the vascular supply (*N*_*s*_), eventually approaches the neuronal energy demand (*N*_*d*_). Here, *N*_*s*_ is given by the summation of the vessel states as described in Equation 14. The tissue perfusion levels are reflected by the quantity called the “energy deficit” (*e*), representing the instantaneous difference between the demand and the supply, given in Equation 15.

(14) $$\begin{array}{l} N_{s}=\overset{n}{\underset{j=1}{\sum }}S_{j} \end{array}$$

(15) $$\begin{array}{l} e=N_{d}-N_{s} \end{array}$$

(16) $$\begin{array}{l} \tau_{e}\frac{dE}{dt}=\tanh\left(\lambda_{e}e\right) \end{array}$$

(17) $$\begin{array}{l} I=E-\frac{n}{2} \end{array}$$

Here, *E* is the accumulated deficit, τ_*e*_ is the time constant for the accumulation and *I* is the final deficit signal coming to the vessels, representative of the vasoactive mediators, released by the neuron-glial system, and control the vascular dynamics according to the energy deficit, *e*.

### 2.3. Neurovascular coupling

The objective of the auto-encoder coupled vascular network is to minimize the output reconstruction error (*mse*). Srivastava et al. (2014) have shown that a certain percentage of dropout (*q*) in the hidden layer of the auto-encoder helps minimize *mse*. In the present study, *q* in the hidden layer is governed by the percentage of inactive (OFF) vessels in the vascular network, which in turn is dictated by neuronal energy demand (*N*_*d*_). The change in *mse* is fed to the vascular system in order to update *N*_*d*_.

**Figure 2:**
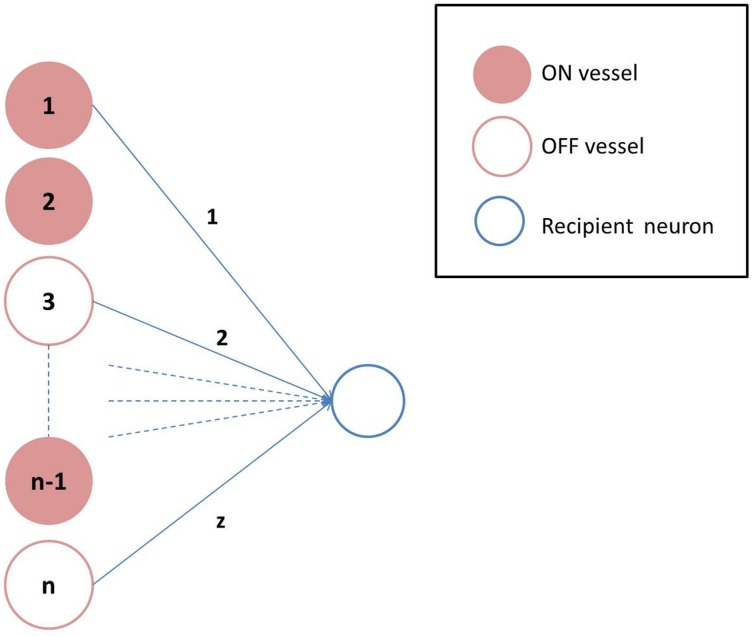
**Vascular projections to a single neuron: Each neuron receives vascular inputs from *z* of the *n* vessels**. The neuron would be in the ON state, if the average activity of the *z* vessels is positive as described in Equation 19.

The *N*_*d*_ is initialized at the maximum possible value, implying that all the vessels are ON, corresponding to *q* = 0. The value of *N*_*d*_ is updated with respect to *mse*, as given by Equation 18.

(18) $$\begin{array}{l} N_{d}\left(t+1\right)=N_{d}\left(t\right)-\beta \frac{\Delta mse}{\Delta N_{d}} \end{array}$$

where, β is a model parameter that represents the rate of perfusion. Depending on the value of *N*_*d*_, the states of individual vessels could be ON/OFF. The connectivity between the neuronal and the vascular layer determines the states (ON/OFF) of the individual neurons in the hidden layer of the auto-encoder. Formally, each neuron could receive inputs from any *z* of the *n* vessels, chosen randomly, with each vessel's state scaled by a factor of 1∕*z*. Thus, the *r*_*i*_ (an inverse correlate of neuronal firing threshold) corresponding to each unit in the hidden layer is given by Equation 19 and determines whether that neuron is OFF (dropped out) or not, as described previously in Equation 6.

(19) $$\begin{array}{l} r_{i}=H\left(\frac{1}{z}\overset{n}{\underset{j=1}{\sum }}a_{ij}S_{j}\right) \end{array}$$

(20) $$\begin{array}{l} a_{ij}=\left\{ \begin{array}{cc} 1\; & \;:\text{ith neuron is connected to }j\text{th vessel}\\ 0\; & :\text{ith neuron is not connected to }j\text{th vessel} \end{array}\right. \end{array}$$

(21) $$\begin{array}{l} \overset{n}{\underset{j=1}{\sum }}a_{ij}=z \end{array}$$

where **H** is the Heaviside Step function; and *a*_*ij*_ represents the connectivity between the *i*th neuron and *j*th vessel. A schematic representation of the vascular projections to a single neuron is illustrated in Figure 2 If *N*_*d*_ is too large, *mse* will increase as the dropout percentage is too low. On the contrary, if *N*_*d*_ is too low, due to “over-sparseness,” *mse* will increase. Hence, the objective of Equation 18 is to search for the optimal *N*_*d*_ that minimizes *mse*. The essence of the neurovascular coupling lies in the symbiosis between *N*_*d*_ and *mse*.

## 3. Experiments and results

In this section, some of the prominent simulation results are described, on implementation of the conceptualized model. These results can be summarized as follows:

- Desynchronized vascular dynamics enables the auto-encoder network to learn sparse independent features, by introducing stochastic dropout in its hidden layer.
- The connectivity pattern between the neural and the vascular network governs the stochasticity in the hidden layer. Thus, by itself, desynchronized vascular dynamics do not ensure sparse independent features being learnt.
- The time scale of the vascular dynamics has a direct bearing on the temporal stochasticity in the hidden layer.

The auto-encoder network efficiency is described by its ability to learn sparse features. Two datasets are used to train the network, highlighting the importance of the desynchronized vascular activity in extracting sparse independent features. The first data set consists of 5000 images of size 8 × 8, each of which contains a random number of horizontal (1 × 8) or vertical (8 × 1) bars. For this data set it is fairly easy to speculate as to how independent weight patterns should appear. If 16 neurons are chosen in the hidden layer, each of them should respond to a single bar (either vertical or horizontal) in order to achieve a sparse representation of the input bar patterns. A similar data set has been used by Földiak (1990) to study evolution of sparse features. In the second data set, the MNIST digit data, it is not quite apparent how the independent components of the input data should appear. The MNIST digit dataset consists of 60,000 images of size 28 × 28, each of which contains a single handwritten digit. The performance of the model is assessed on the basis of three performance measures: converged value of *mse*, mutual information index (MII), and structural similarity index (SSI). MII is used to measure the interdependencies between a pair of images (here weight patterns) and is given by Equation 22 (Mora and Ucelay, 2009).

(22) $$\begin{array}{l} MII=S\left(X\right)+S\left(Y\right)-S\left(X,Y\right) \end{array}$$

where, *S*(*X*) and *S*(*Y*) are the entropies calculated on the histograms of the individual values of pixels in the two images X and Y, respectively; *S*(*X, Y*) represents the joint entropy, similarly calculated from the joint histogram of *X* and *Y*. Thus, for sparse independent weights, MII would be low. On the contrary, the non-sparse weights would have high MII, signifying more overlap in the information being conveyed by them. The third measure for evaluating the network is SSI that measures the qualitative similarity between a pair of images (Wang et al., 2004). SSI is a multiplicative combination of three terms: the luminance term, the contrast term and the structure term for the two images (X and Y) as given by Equation 23.

(23) $$\begin{array}{l} SSI=\frac{\left(2\mu \left(X\right)\mu \left(Y\right)+C_{1}\right)\left(2\sigma \left(X,Y\right)+C_{2}\right)}{\left(\mu {\left(X\right)}^{2}+\mu {\left(Y\right)}^{2}+C_{1}\right)\left(\sigma {\left(X\right)}^{2}+\sigma {\left(Y\right)}^{2}+C_{2}\right)} \end{array}$$

where μ(*X*) and μ(*Y*), σ(*X*) and σ(*Y*) and σ(*X, Y*) represent the means, standard deviations and cross covariance for the images *X* and *Y*, respectively. *C*_1_ and *C*_2_ are parameters of the equation. In the present context, SSI gives a measure of the sparseness, as conceptually it is based on the idea that spatially close pixels carry information of the structures in the image. Hence, for sparse independent features, SSI should be high as compared to the non-sparse weight patterns.

### 3.1. Impact of vascular dynamics: synchronized or desynchronized

**Figure 3:**
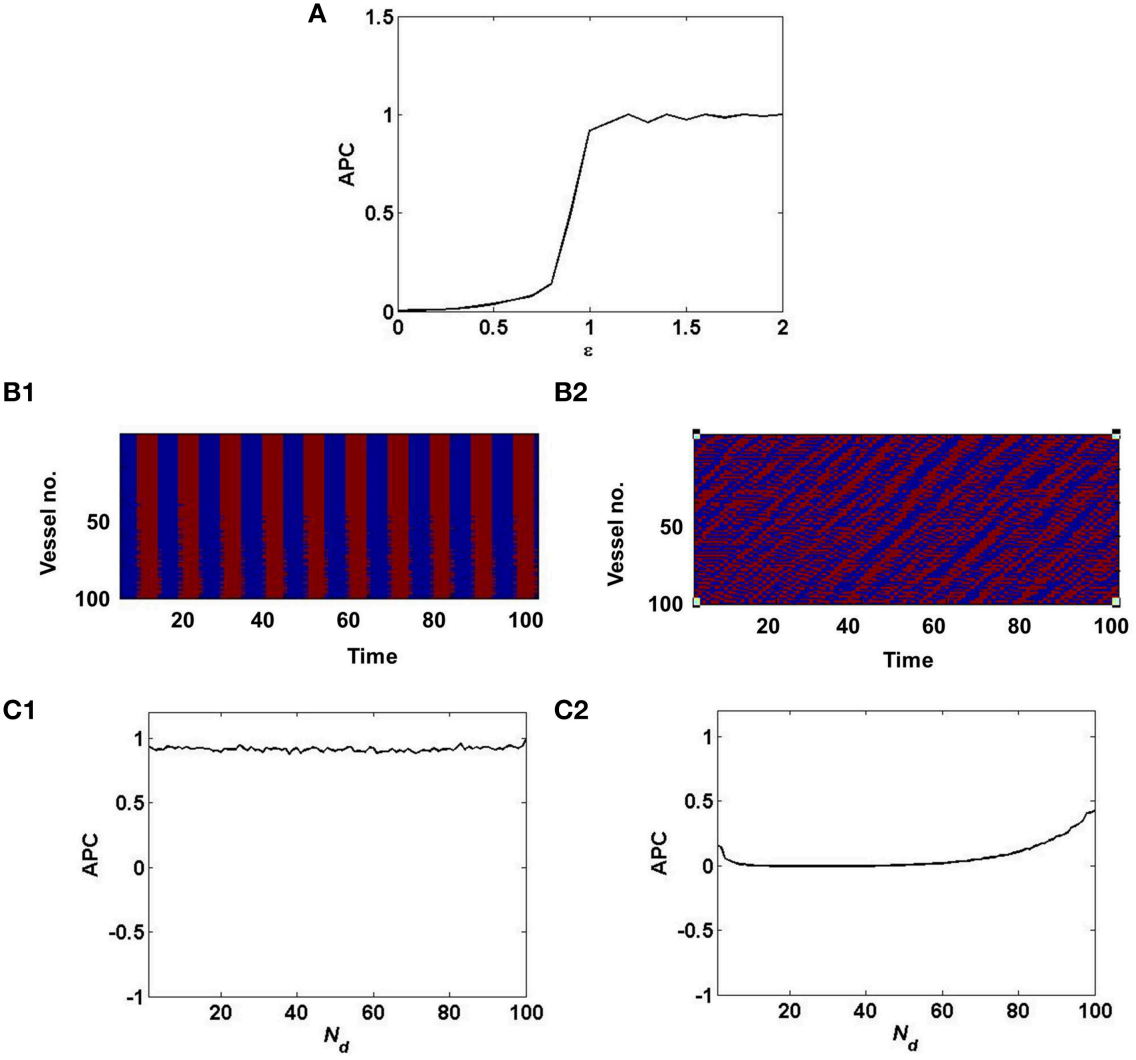
**Synchronized vessel activity with individual periodic dynamics; compared with desynchronized vessel activity with individual chaotic dynamics**. **(A)** depicts the measure of synchrony, APC for different values of ϵ; **(B1)** Synchronized activity seen among 100 vessels across time; **(B2)** Desynchronized activity seen among 100 vessels across time. **(C1)** APC for different values of *N*_*d*_, assuming synchrony (ϵ = 1); **(C2)** APC for different values of *N*_*d*_, assuming desynchrony (ϵ = 0).

The internal connectivity pattern within the vascular network determines the degree of synchronization amongst the individual vascular units. Synchrony among the vessels in the vascular network is characterized by a parameter: Average Pairwise Correlation (APC). The correlation (γ_*jk*_) between a pair of vessel states (*S*_*j*_, *S*_*k*_) over time is given by Equation 24. The averaged correlation over all such pairs of vessels gives the APC (Equation 25).

(24) $$\begin{array}{l} \gamma_{jk}=\frac{\sum_{t}\left(S_{j}\left(t\right)-\bar{S_{j}}\right)\left(S_{k}\left(t\right)-\bar{S_{k}}\right)}{\sqrt{\sum_{t}{\left(S_{j}\left(t\right)-\bar{S_{j}}\right)}^{2}\sum_{t}{\left(S_{k}\left(t\right)-\bar{S_{k}}\right)}^{2}}} \end{array}$$

(25) $$\begin{array}{l} APC=\frac{1}{n\left(n-1\right)}\overset{n}{\underset{j}{\sum }}\overset{n}{\underset{k\neq j}{\sum }}\gamma_{jk} \end{array}$$

where, *n* is the number of vessels and *n*(*n* − 1) is the number of pairs of distinct vessels. As described in Equation 12, decreasing the value of ϵ, switches the dynamics of the vascular network from synchronized to desynchronized as quantified by the APC (Figure 3). This in turn governs the stochasticity in the hidden layer of the coupled auto-encoder. The dropout in the hidden layer is governed by the states (*S*_*j*_) of individual vessel units.

In the case of desynchronized vascular dynamics, assuming one-to-one connectivity with the hidden layer, each vessel units activity turns ON/OFF neurons in the hidden layer in a random fashion. Randomized dropout is crucial in order to minimize *mse* and learn sparse independent features. The randomness of the dropout of neurons in the hidden layer, is introduced by the desynchronized vascular input.

Now when the hidden layer receives synchronous vascular input, assuming one-to-one connectivity with the hidden layer, all of the hidden neurons are either turned ON/OFF in a particular learning iteration. Hence the randomness of dropout is lost. Thus, when all the hidden neurons are ON, there is no dropout, hence no sparse independent features learnt; when all the hidden neurons are OFF, there is no learning, since the corresponding weight values will not be updated.

**Figure 4:**
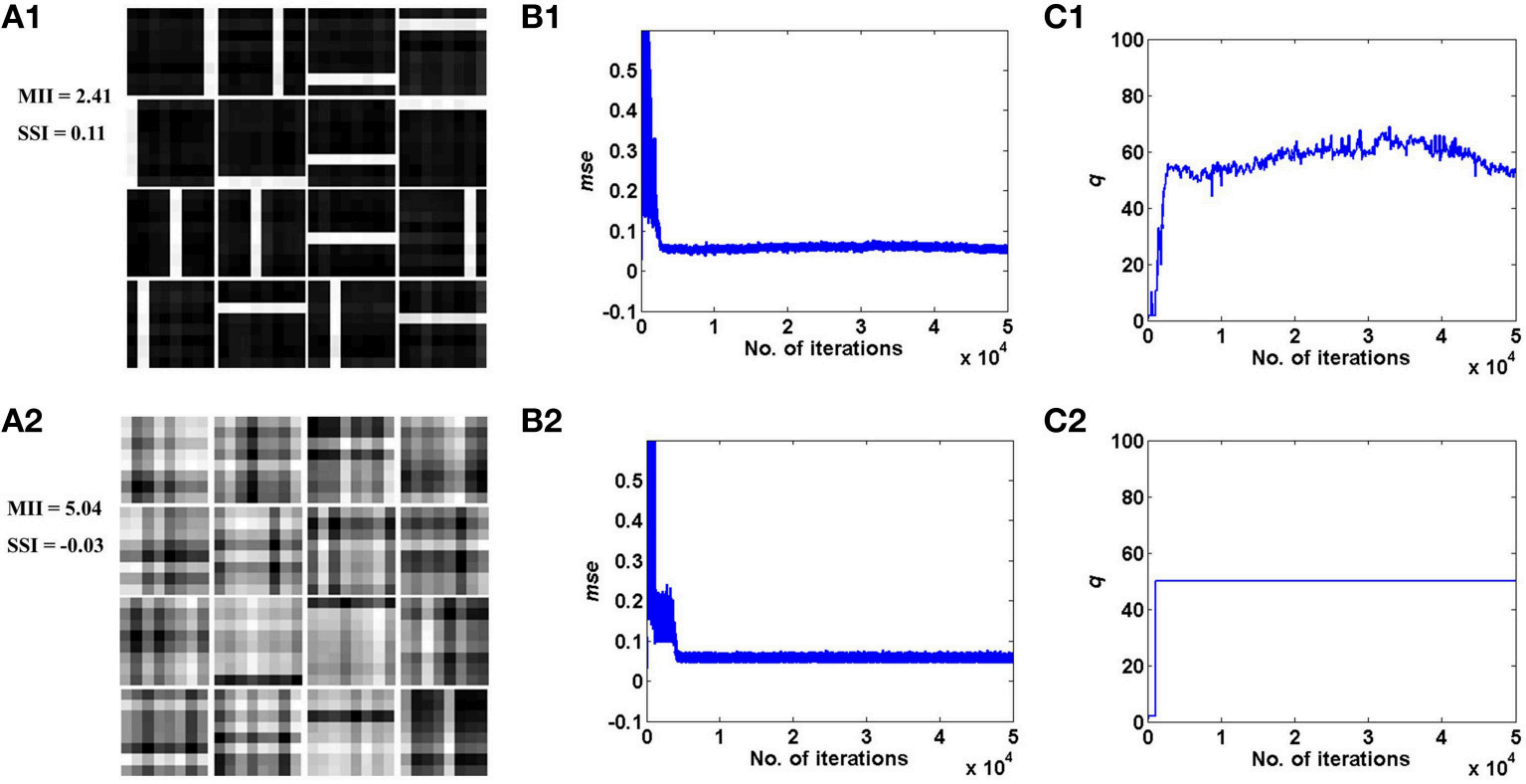
**Desynchronized (ϵ = 0) vascular dynamics, assuming a one-to-one connectivity with the neurons in the hidden layer, results in sparse independent features being learnt (top row), as opposed to synchronized (ϵ = 1) vascular dynamics (bottom row), on training on bar patterns**. **(A1,A2)** depict the weight patterns learnt by the neurons in the hidden layer under desynchronized and synchronized vascular dynamics, along with their independence measures MII and SSI; **(B1,B2)** demonstrate that the final converged value for *mse* is lower (0.055) under the influence of desynchronized vascular activity as compared to synchronized (0.062); **(C1,C2)** shows the trend of the percentage of dropout (*q*) of neurons in the hidden layer.

**Figure 5:**
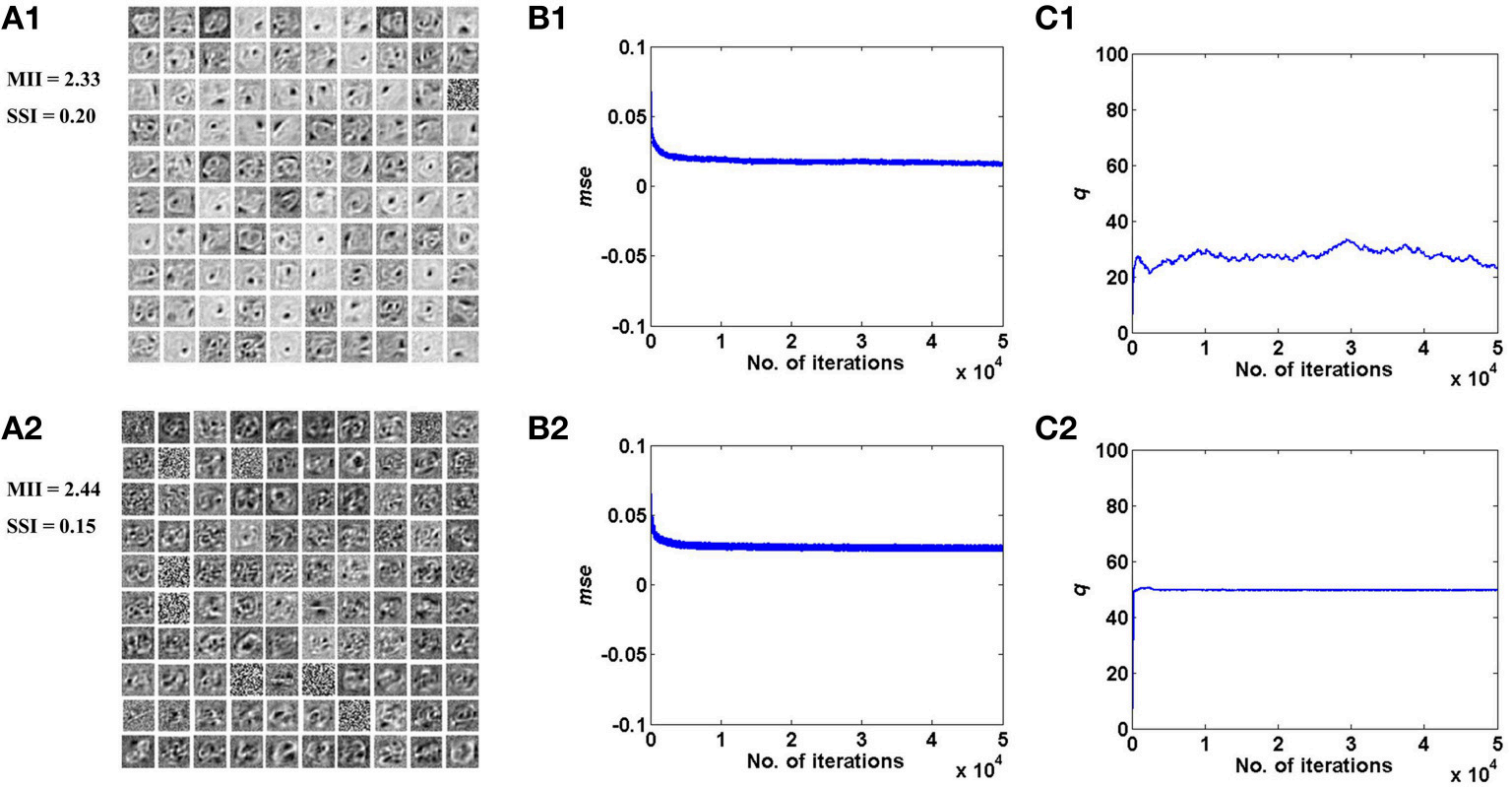
**Desynchronized (ϵ = 0) vascular dynamics, assuming a one-to-one connectivity with the neurons in the hidden layer, results in sparse independent features being learnt (top row), as opposed to synchronized (ϵ = 1) vascular dynamics (bottom row), on training on the MNIST digit data**. **(A1,A2)** depict the weight patterns learnt by the neurons in the hidden layer under desynchronized and synchronized vascular dynamics, along with their independence measures MII and SSI; **(B1,B2)** demonstrate that the final converged value for *mse* is lower (0.016) under the influence of desynchronized vascular activity as compared to synchronized (0.026); **(C1,C2)** shows the trend of the percentage of dropout (*q*) of neurons in the hidden layer.

These results are illustrated in Figures 4, 5 for the bar and MNIST datasets respectively. The first row in both figures corresponds to the hidden layer receiving desynchronized vascular input; whereas the bottom row corresponds to synchronized case. The features learnt under desynchronized vascular input are sparse and independent as compared to synchronized, quantified by their MII and SSI scores. It is also observed that the *mse* for both data sets is minimized in the case of desynchronized vascular input compared to synchronized input (desynchronized: 0.055, synchronized: 0.062, for the bar dataset; desynchronized: 0.016, synchronized: 0.026, for the MNIST dataset).

### 3.2. Impact of the spatial parameter: vascular-neural connectivity

The connectivity scheme between the vascular and the neural network is as important as the vascular intralayer connectivity. The complete range of connectivity configurations between the two layers are considered. If each neuron receives an input from a single vessel, it is termed as one-to-one connectivity, as mentioned in the previous subsection. On the other hand, if each neuron receives inputs from every vessel a complete connectivity scheme is achieved. It is obvious that on complete connectivity, the stochasticity introduced due to desynchronized activity of the vessels is lost, as each neuron receives exactly the same feedback. Thus, the neural network's ability to learn sparse features is gradually degraded on traversing from a one-to-one toward an all-to-all connectivity architecture.

**Figure 6:**
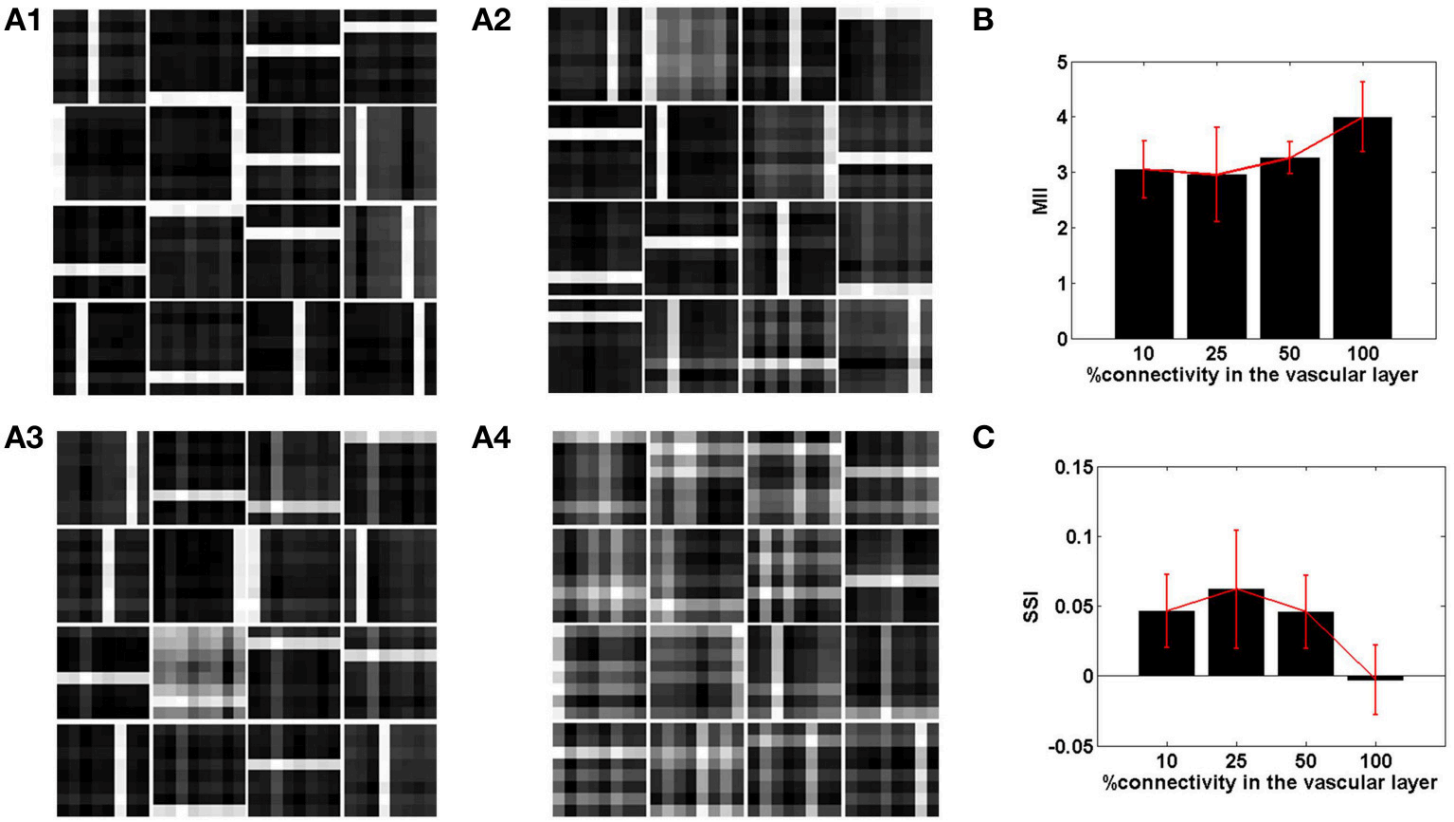
**The role of the spatial parameter (vascular-neural connectivity), on learning sparse independent features, assuming desynchronized vascular activity (ϵ = 0 and APC ≊ 0), when trained on bar patterns**. **(A1–A4)** are representative of the learnt weight matrices of neurons in the hidden layer on increasing the projective field from one-to-one (*k* = 1) to complete (*k* = *n*) connectivity; **(B)** demonstrates that the MII shows an upward trend on average on increasing the projective field from one-to-one (*k* = 1) to complete (*k* = *n*) connectivity; **(C)** demonstrates that the SSI shows a downward trend on average on increasing the projective field from one-to-one (*k* = 1) to complete (*k* = *n*) connectivity.

**Figure 7:**
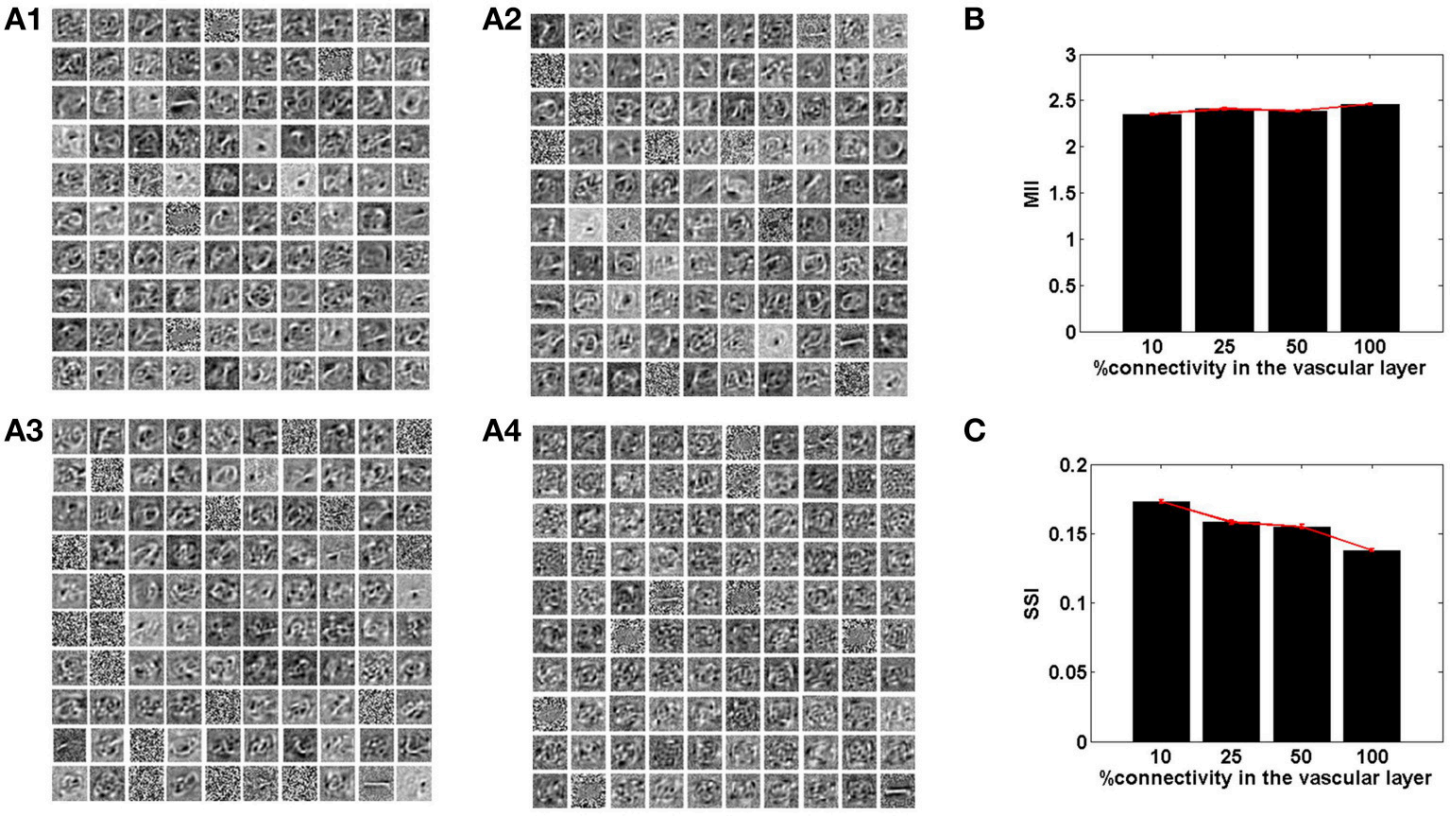
**The role of the spatial parameter (vascular-neural connectivity), on learning sparse independent features, assuming desynchronized vascular activity (ϵ = 0 and APC ≊ 0), when trained on MNIST digit data**. **(A1–A4)** are representative of the learnt weight matrices of neurons in the hidden layer on increasing the projective field from one-to-one (*k* = 1) to complete (*k* = *n*) connectivity; **(B)** demonstrates that the MII shows an upward trend on average on increasing the projective field from one-to-one (*k* = 1) to complete (*k* = *n*) connectivity; **(C)** demonstrates that the SSI shows a downward trend on average on increasing the projective field from one-to-one (*k* = 1) to complete (*k* = *n*) connectivity.

The sparse independent features learnt, on differing degrees of interlayer connectivity are shown in Figures 6, 7, along with their corresponding performance measures. Assuming the vascular input is desynchronized, for various connectivity patterns (10, 25, 50, 100% connectivity), the features learnt are shown. On increasing the projective field, the randomness of dropout in the hidden layer is lost and as a result the features learnt are no longer sparse and independent. This is verified using the two measures: MII and SSI.

### 3.3. Impact of the temporal parameter: neural-vascular time-scale ratio

In the context of the present model that studies the effects of neurovascular coupling, the ratio of time scales of the neural and vascular dynamics assumes significance. The vascular time step (*dt*) describes the rate at which vascular dynamics are updated. The temporal ratio (TR) is defined as the number of input patterns presented to the neuronal layer during a single vascular time step. Therefore, TR denotes the ratio of time scales of stimulus presentation to the neural network vs. vascular dynamics.

**Figure 8:**
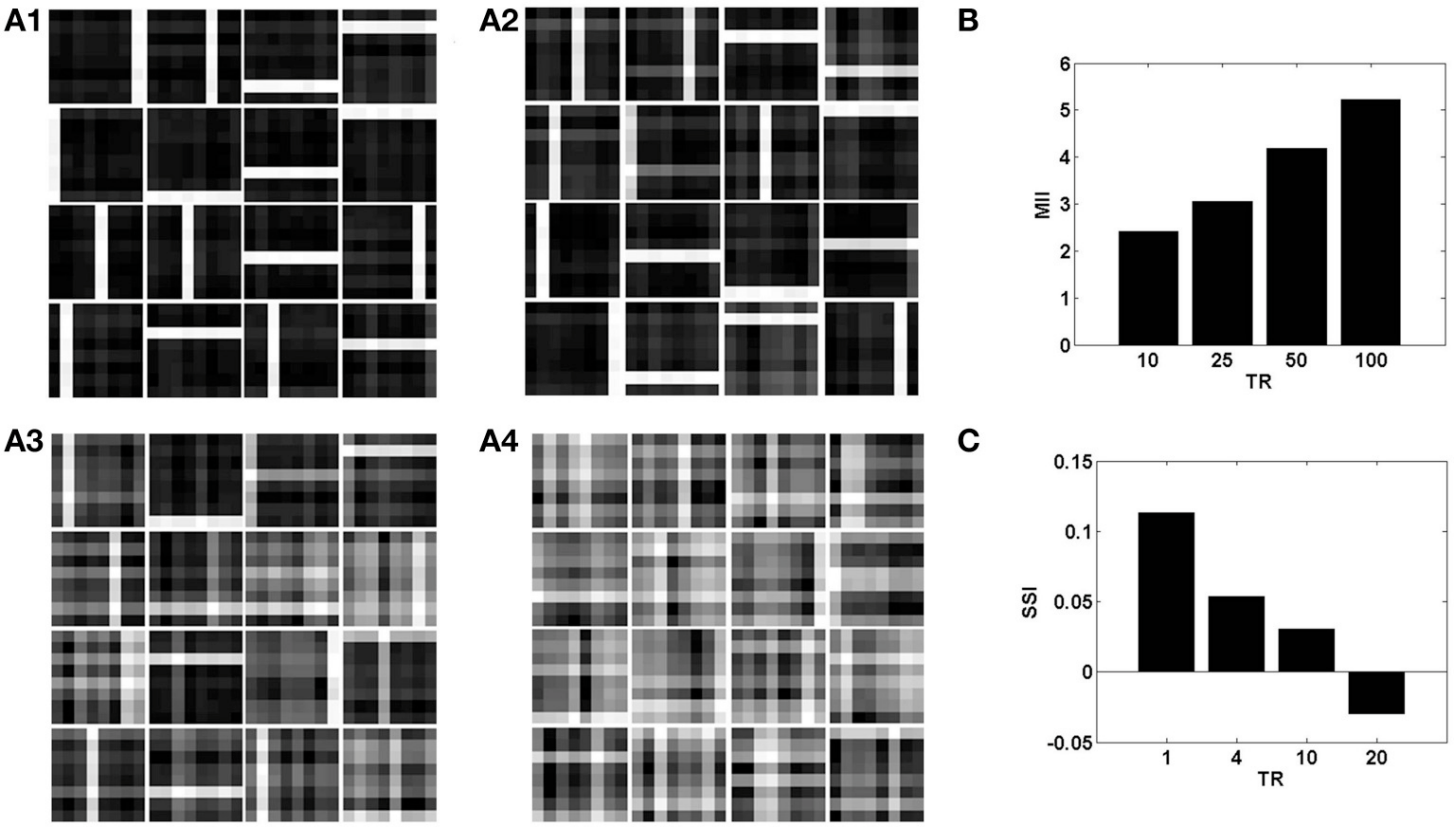
**The role of the temporal parameter (TR), on learning sparse independent features, assuming desynchronized vascular activity (ϵ = 0 and APC ≊ 0), when trained on bar patterns with *k* = 1**. **(A1– A4)** are representative of the learnt weight matrices of neurons in the hidden layer on increasing the time delay; **(B)** demonstrates that the MII shows an upward trend on increasing the temporal ration (TR); **(C)** demonstrates that the SSI shows a downward trend on increasing the temporal ratio (TR).

**Figure 9:**
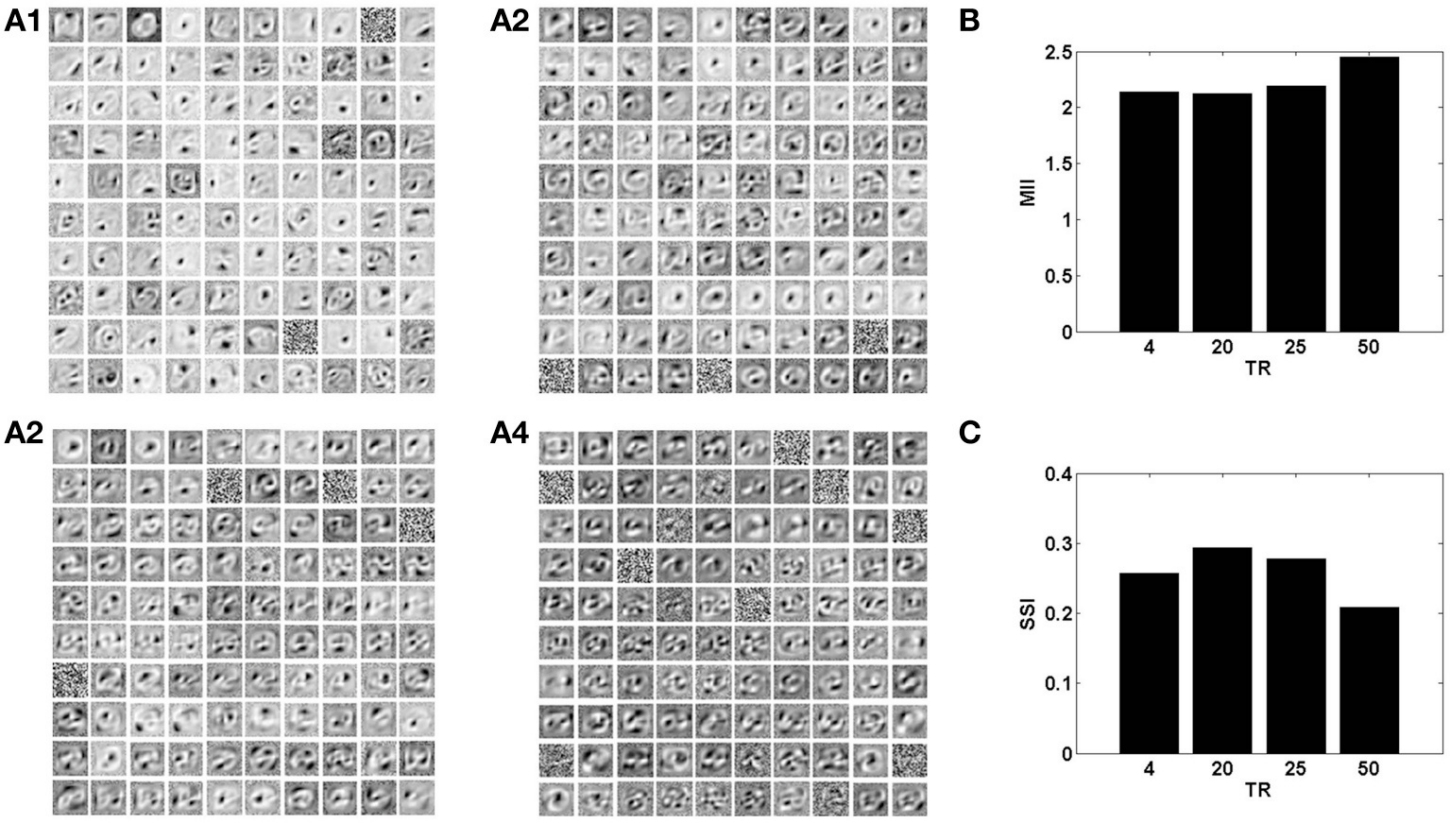
**The role of the temporal parameter (TR), on learning sparse independent features, assuming desynchronized vascular activity (ϵ = 0 and APC ≊ 0), when trained on MNIST digit data with *k* = 1**. **(A1–A4)** are representative of the learnt weight matrices of neurons in the hidden layer on increasing the time delay; **(B)** demonstrates that the MII shows an upward trend on increasing the temporal ratio (TR); **(C)** demonstrates that the SSI shows a downward trend on increasing the temporal ratio (TR).

With a lower TR, it was observed that the network shows better performance for both datasets, as illustrated by the performance measures; MII and SSI in Figures 8, 9. On the contrary, increasing the TR adversely affects the learning of the sparse independent features. Figures 8, 9 depict that with a higher TR, the network efficiency becomes equivalent to that observed in the case of synchronized vascular dynamics. This is due to the fact that for large values of TR, the same sets of neurons in the hidden layer are dropped out for a number of input presentations. As a result the randomness of dropout is compromised, leading to larger reconstruction errors. Thus, these results suggest that the TR should be optimal for efficient information representation.

## 4. Discussion

The present study proposes a neural network model in which modulatory feedback from vascular dynamics has a direct influence on learning and performance of the neural network. Most neurovascular models focus on the feedforward effect of neural dynamics on vascular dynamics, and ignore the feedback influence of vascular dynamics on neural activity. Drawing from experimental literature, a strong case for the significant influence of vascular feedback on neural activity has been theorized by Moore and Cao (2008). Interestingly, a number of cognitive impairments have been associated with vascular dysfunctions (Di Marco et al., 2015). The proposed model specifically emphasizes the role of the vascular system in information representation in neural circuits. Within an auto-encoder framework, the influence of desynchronized vascular dynamics on the information representation in a neural network is investigated. It is further demonstrated that the spatio-temporal characteristics of the vascular network must be optimal for the neural network to learn efficient, sparse independent representations of input data.

The model impinges on the capability of the vascular system to introduce a certain percentage of dropout in an auto-encoder. It has previously been demonstrated that dropout in the hidden layer is imperative for learning sparse independent features (Srivastava et al., 2014). In the present study, the stochastic dropout has been linked to desynchronized vascular dynamics, given the requisite temporal and spatial parameters. The desynchronized vascular dynamics are a result of the vascular intra-connectivity matrix (T). Furthermore, it has been demonstrated that one-to-one connectivity between the neural and the vascular layer, is adequate in bringing about the requisite stochasticity in the neuronal layer, given desynchronized vascular dynamics. The spatial parameter, k, represents the size of the projective field of individual vascular units onto the neuronal layer. Experimental studies report that the ratio of the number of neurons to capillaries is 1:1, suggesting that a one to one connectivity between the neural and vascular layer is feasible (Quaegebeur et al., 2011). In addition, with intercapillary distances of 40 μ*m*, each capillary supplies neurons within a radial distance of 25 μ*m* (Pardridge, 2011). However, it is possible that due to the dynamic nature of the blood flow, this radius of influence could be larger and of the radius of astrocytic microdomains ≈ 100 − 200 μ*m* (Oberheim et al., 2006). Under the assumption of these larger radii of vascular influence, the one-to-one relation between vessels and neurons is no longer valid. Each neural node in the model could now however be reinterpreted as a set of neurons having similar receptive fields as in a cortical column, rather than a single neuron. Thus, two nodes in the hidden layer would represent two non-overlapping cortical receptive field domains which are known to be ≈ 500μ*m* apart (Leise, 1990).

The neural network model considered in this paper is rate coded. This implies that the value of a particular node in the network signifies the firing rate of that node. Thus, when the vascular dynamics results in the dropout of a particular node, it implies that for the entire duration of the corresponding vessels' cycle, that particular node is silent. The neuronal weight update is of the order of the timescale of vascular dynamics. Also it must be noted that the time scale of vascular dynamics is of the order of stimulus presentation. These points lead to the testable hypothesis: Assuming the time for a response spike train to be insignificant as compared to the vascular dynamics, how many separate input stimuli could be presented within a single vascular cycle, such that the performance of the network is not compromised? The temporal ratio (TR) is intended to help arrive at this number.

The role of vascular plasticity is not elaborated in the current model. It is known that angiogenesis follows new learning via the expression of Vascular Endothelial Growth Factor (VEGF) within minutes of changes in oxygen demands in the brain (Welberg, 2011). In the model described, post training, each of the neuronal hidden nodes respond on the presentation of at least one of the input stimuli considered, and hence are active at different time points corresponding to their presentation. The frequency of activation of different nodes is close to uniform when considered across input presentations due to the nature of the stimuli set considered. This implies that though the overall network demand changes, it changes equally for each of the individual neurons. In the light of this, for the model in its current formulation, uniform connections from the vascular layer to the hidden neuronal layer should be sufficient, and angiogenesis, which implies, among other things, training of connections from the vascular layer to the hidden layer, is not necessary. However, when the input stimuli are skewed, angiogenesis, expressed in the form of trainable vascular-neural connections, will become important.

A number of recent experimental findings corroborate that predictive coding mechanisms are at play in the visual system (Allman et al., 1985; Miller et al., 1991; Spratling, 2008; Egner et al., 2010). Such a predictive coding model has been shown to learn independent features based on the overall image statistics. However, additional requirements such as a hierarchy of such predictive modules, sparsity constraints, and predefined image patches need to be employed to extract meaningful features (Rao and Ballard, 1999). In the predictive coding framework, the ensemble input reconstruction error (*mse*) represents the activity of the error estimating neural layer. Traditionally, this error is employed to change the synaptic weights and biases associated with the network, such that the prediction mimics reality (Hinton and McClelland, 1988). In the present model, *mse* additionally influences the feedforward signal from the neural to the vascular layer. The rationale for utilizing this lumped *mse*, is pivoted by experimental evidence suggesting that neurons work in concert to regulate vascular dynamics (Cohen et al., 1997; Krimer et al., 1998). The activity of the error estimating neurons is fed to the vasculature associated with the hidden layer neurons in the model. This phenomenon of distal neuronal control of the vascular dynamics is a known mechanism of neural regulation of cerebral blood flow (Cohen et al., 1997; Iadecola, 1998; Krimer et al., 1998). The change in *mse* is interpreted by the vascular system as the neural demand (*N*_*d*_). The vascular system optimizes its supply such that at every time instant the neural demand is met. The optimization paradigm is similar to that implemented by Pradhan and Chakravarthy (2007) in the context of perfusion in skeletal muscle.

In general, cerebral blood vessels show spontaneous oscillations known as vasomotion in physiological conditions, which can either be chaotic or periodic (Griffith and Edwards, 1994). “Chaos,” in physiological systems, is considered to provide certain inherent variability, characteristic of normal physiology, while the loss of such variability is a sign of impaired health (Nilsson and Aalkjaer, 2003). Experimental studies by Griffith (1996) depict the link between temporal chaos in microvasculature and efficient energy delivery. However, in the present context it is more relevant to consider vasomotion as a network phenomenon, in lieu of fluctuations in the lumen of a single, isolated capillary. This is because the vascular dynamics is largely governed by complex interactions among multiple interconnected capillaries communicating through physicochemical signals. Thus, the significance of vasomotion rhythms (regular or chaotic) is more naturally appreciated in terms of the dynamics of microvascular network (Pradhan and Chakravarthy, 2011). A previous modeling study highlighted the link between desynchronized vascular dynamics and efficient energy delivery, in skeletal muscles (Pradhan et al., 2007; Pradhan and Chakravarthy, 2009). In the current framework, it is shown that desynchronized vascular dynamics are essential for efficient training. Desynchronized vascular activity, as currently modeled, could be considered a consequence of chaotic capillary dynamics.

Physiologically, could such desynchronized dynamics have an importance in information representation in an associated neural tissue, in addition to simply allowing efficient energy delivery? While there is meager biological evidence to second the association of desynchronized vascular dynamics with information processing, there are studies suggesting the presence of periodic vasomotion under metabolically compromised states such as hypoxia and ischaemia (Nilsson and Aalkjaer, 2003). Such conditions have been shown to result in impaired neural plasticity (Failor et al., 2010). These two studies predict a crucial link between the prevalence of vasomotion and plasticity in the brain. However, the precise causal relationships between abnormal vasomotion and cognitive dysfunctions need to be experimentally explored further. Furthermore, it would be interesting to understand the correlation of the different frequencies and amplitude of vasomotion on learning and plasticity in the brain.

Although the present model is abstract, it preserves the essence of known mechanisms of neurovascular coupling. Moreover, it proposes a novel paradigm of “vascular computation,” wherein the vascular network actively participates in information processing. Within this framework, biologically realistic neurovascular models could be developed, incorporating the biophysical details of neurons and blood vessels. An example of such a detailed framework would include modeling the signaling pathways from the neurons to the vessels leading to dilation/constriction. The downstream effect of this further can be reflected in the glucose, lactate and oxygen release probabilities from the vessels, as was described by Chander and Chakravarthy (2012). The influence of these energy substrates on the neuron could then be modeled in a similar fashion as that of the present study via controlling the neural firing threshold. Moreover, there are significant evidences suggesting an energy dependent control of the firing threshold through the activity of the *K*_*ATP*_ channels (Ballanyi, 2004). It would not be unreasonable to expect such a detailed model to manifest all the underlying features of the abstract model described in this paper.

Another plausible attempt would be to consider a vascular anatomic network model, proposed by Boas et al. (2008) along with detailed spiking neuron models. The neural network considered in this paper is rate coded, hence sub-threshold neuronal oscillation which could influence vascular dynamics are not considered. Such approaches might help in establishing the informational association between the BOLD and EEG signals. Further studies need to be pursued in the path paved by the model in order to precisely understand the plasticity within the neuronal and vascular networks.

## Author contributions

KC: Computational model development, analysis and manuscript preparation; RTP: Computational model development, analysis and manuscript preparation; VSC: Computational model development, analysis and manuscript preparation.

### Conflict of interest statement

The authors declare that the research was conducted in the absence of any commercial or financial relationships that could be construed as a potential conflict of interest.
